# Anti-*Neospora caninum* and anti-*Toxoplasma gondii* antibodies in cattle intended for human consumption in the State of Espírito Santo, Brazil: prevalence and associated factors

**DOI:** 10.1590/S1984-29612025026

**Published:** 2025-06-02

**Authors:** Samira Pereira Batista, Jordania Oliveira Silva, Carolina Magri Ferraz, João Pedro Barbosa de Assis, Gabriel Augusto Marques Rossi, Luiza Guilherme Daleprani, Fernando Luiz Tobias, Thais Ferreira Feitosa, Fábio Ribeiro Braga, Vinícius Longo Ribeiro Vilela

**Affiliations:** 1 Programa de Pós-Graduação em Ciência e Saúde Animal, Universidade Federal de Campina Grande, Patos, PB, Brasil; 2 Departamento de Medicina Veterinária, Instituto Federal de Educação, Ciência e Tecnologia da Paraíba, Sousa, PB, Brasil; 3 Laboratório de Microbiologia e Imunologia Veterinária, Universidade Vila Velha – UVV, Vila Velha, ES, Brasil

**Keywords:** Neosporosis, ruminants, seropositivity, toxoplasmosis, Neosporose, ruminantes, soropositividade, toxoplasmose

## Abstract

The objective of this study was to describe the seroprevalence and factors associated with *Neospora caninum* and *Toxoplasma gondii* infections in cattle in the state of Espírito Santo, Brazil. Serum samples were collected from 600 slaughtered cattle and destined intended for consumption, originating from 27 municipalities. The samples were analyzed using the Indirect Immunofluorescence Test, with cut-off points for anti-*N. caninum* antibodies at 1:200 and for anti-*T. gondii* antibodies at 1:64. Positive samples were titrated until negative. Epidemiological questionnaires were applied to gather information about the properties and herds. The seroprevalence for *N. caninum* was 13.2% (79/600), with antibody titers ranging from 1:200 to 1:6400, and for *T. gondii* it was 12.5% (75/600), with titers ranging from 1:64 to 1:1024. In the multivariate analysis, the factor associated with *N. caninum* infection was a history of abortion (OR 6.483; CI 6.082-6.884; p < 0.01), and for *T. gondii*, contact with cats (OR 7.683; CI 7.172-8194; p < 0.005). The seroprevalences for *N. caninum* and *T. gondii* in cattle slaughtered for human consumption in Espírito Santo are significant, with a history of abortion being a factor associated with *N. caninum* infection and contact with cats associated with *T. gondii* infection.

## Resumo

Objetivou-se descrever a soroprevalência e os fatores associados às infecções por *Neospora caninum* e *Toxoplasma gondii* em bovinos no Estado do Espírito Santo, Brasil. Foram coletadas amostras de soros de 600 bovinos abatidos e destinados ao consumo, provenientes de 27 municípios. As amostras foram analisadas por Reação de Imunofluorescência Indireta, com pontos de corte para anticorpos anti-*N. caninum* de 1:200 e para anticorpos anti-*T. gondii* de 1:64. Amostras positivas foram tituladas até negativação. Foram aplicados questionários epidemiológicos para acessar informações referentes às propriedades e aos rebanhos. A soroprevalência para *N. caninum* foi de 13,2% (79/600), com títulos de anticorpos variando de 1:200-1:6400. E para *T. gondii* foi de 12,5% (75/600), com títulos variando de 1:64-1:1024. Na análise multivariada, o fator/ consequência associada à infecção por *N. caninum* foi o histórico de aborto (OR 6.483; CI 6.082-6.884; p < 0,01) e para *T. gondii*, o contato com gatos (OR 7.683; CI 7.172-8194; p < 0,005). São expressivas as soroprevalências para *N. caninum* e *T. gondii* em bovinos abatidos para o consumo humano no Estado do Espírito Santo, sendo o histórico de aborto um fator associado à infecção por *N. caninum* e o contato com gatos à infecção por *T. gondii*.

## Introduction

Neosporosis and toxoplasmosis are coccidian infections that can affect ruminants and other vertebrate animals, generally causing reproductive and neurological issues, but they can also affect other systems, such as the hepatic, cardiac, and renal systems (Varaschin et al., 2012; González-Warleta et al., 2014; Ferreira et al., 2018; Malta et al., 2019; Didiano et al., 2020; Perotta et al., 2021). In ruminants, the transmission of these diseases can occur horizontally, through the ingestion of oocysts in contaminated food or water, and vertically, through the transmission of tachyzoites from the mother to the fetus (Dubey et al., 1970; Dubey, 2009). Although both modes of transmission are important for the occurrence of these diseases in herds, vertical transmission is the main via for maintaining infection in herds because it occurs independently of the presence of oocysts in the environment (Costa et al., 2011; Robert-Gangneux, 2014; Diniz et al., 2019).

Cattle are the most susceptible intermediate hosts to infections caused by *Neospora caninum*, a protozoan responsible for neosporosis, with domestic and wild canids as its definitive hosts (Lindsay et al., 1999; Dubey et al., 2011; Varaschin et al., 2012; González-Warleta et al., 2014; Perotta et al., 2021). In cattle, neosporosis leads to decreased reproductive performance, impacting animal health and the financial stability of farms (Bruhn et al., 2013; Reichel et al., 2013; Lefkaditis et al., 2020; Santos et al., 2020). Immunosuppressed animals may experience reproductive issues such as delayed estrus, repeated estrous cycles, and fetal mummification. In immunocompetent animals, neosporosis can be subclinical, with chronically infected yet clinically healthy animals capable of transmitting the parasites to subsequent generations (Bruhn et al., 2013; Serrano-Martínez et al., 2019; Lefkaditis et al., 2020).

Toxoplasmosis is a zoonotic disease caused by the protozoan *Toxoplasma gondii*, with domestic and wild felids as its definitive hosts (Dubey et al., 1970; Miller et al., 1972). In humans, toxoplasmosis can cause ocular, reproductive, and neurological complications (Dubey & Jones, 2008; Carmo et al., 2017; Dubey et al., 2020; Dubey, 2021; Kalogeropoulos et al., 2022). In cattle, it is considered a rare disease, likely due to the innate resistance of this species. However, transmission to humans is possible through the consumption of raw or undercooked meat containing tissue cysts (Dubey & Thulliez, 1993; Dubey & Jones, 2008; Shariatzadeh et al., 2021).

It is estimated that the global prevalence of anti-*N. caninum* antibodies in cattle is 20%, with South America having the highest estimated prevalence (24%), equal to the combined estimated prevalences of North and Central America (24%), followed by Asia (18%), Europe (15%), Africa (13%), and Oceania (8%) (Ribeiro et al., 2019). In Brazil, the largest country in South America, seroprevalence varies, ranging from 9.3% in Paraná, 18.1% in Paraíba, 25.4% in Mato Grosso, and 30.2% in Amazonas (Schimidt et al., 2018; Guerra et al., 2019; Azevedo et al., 2021; Maia et al., 2023b). In the Southeast region, where the state of Espírito Santo is located, seroprevalences of 10.6% and 41.6% were observed in São Paulo, and 21.6% in Minas Gerais (Aguiar et al., 2011; Bruhn et al., 2013; Bernardes et al., 2024).

For anti-*T. gondii* antibodies in cattle, the global weighted prevalence was 17.9%. The Americas and Europe are the continents with the highest weighted seroprevalence, at 22.2% and 21.9%, respectively, with values ranging from 0% to 100% (Shariatzadeh et al., 2021). In Brazil, the seroprevalence of anti-*T. gondii* antibodies in cattle also varies across states, such as Goiás (8.5%), Mato Grosso (34.3%), and São Paulo (11.5%) (Maia et al., 2021; Alves et al., 2021; Bernardes et al., 2024).

In the state of Espírito Santo, seroprevalences of anti-*T. gondii* antibodies were found to be 43% in sheep (Acosta et al., 2021) and 46.6% in goats Vicentini et al. (2024). The high seroprevalences observed in small ruminants, along with the similarity in farming methods between these species and cattle, may indicate the possibility of *T. gondii* infection in cattle in the state of Espírito Santo.

Regional serological evaluation is very important for a better understanding of the epidemiology of these diseases (Utuk & Eski, 2019). Thus, the aim of this study was to describe the seroprevalence and factors associated with infections by *N. caninum* and *T. gondii* in cattle slaughtered for human consumption in the state of Espírito Santo, Southeast, Brazil.

## Material and Methods

### Sampling

To determine the number of samples to be analyzed, a randomized sampling calculation was performed according to Thrusfield (2007):


$$n\text{=}\frac{Z^{\text{2}}\times P\left(1-P\right)}{d^{2}}$$ (1)

n= number of samples

Z= normal distribution value for a 95% confidence level

P= expected prevalence of 50%

d= sampling error of 5%

The expected prevalence of 50% was chosen as it represents the scenario of maximum variability, ensuring the most conservative sample size estimation and minimizing the risk of underestimation. The minimum number of samples to be evaluated, as determined by the sampling calculation, was 385. However, for convenience, 600 blood samples were collected from cattle slaughtered for human consumption in the state of Espírito Santo. The samples were collected in pre-labeled glass tubes without anticoagulant to obtain serum.

Blood samples were collected during the exsanguination of cattle on the slaughter line at a slaughterhouse located in the municipality of Fundão, Espírito Santo. The samples were stored in pre-labeled tubes containing a clot activator. After collecting, the tubes were sent to the Veterinary Microbiology and Immunology Laboratory at Vila Velha University, where they were centrifuged at 1.000 g for 10 minutes. Following centrifugation, the sera were extracted using a pipette, transferred to properly labeled microtubes, and kept refrigerated. Subsequently, the samples were sent, under refrigeration, to the Immunology and Infectious Diseases Laboratory at the Federal Institute of Paraíba for analysis.

### Serological diagnosis

For the diagnosis of anti-*N. caninum* antibodies, the Indirect Fluorescence Antibody Test (IFAT) was performed, using a cutoff point of 1:200 (Ghalmi et al., 2012) and following the method described by Dubey et al. (1988). The Nc-1 strains of tachyzoites fixed on slides were used as antigen.

For the detection of anti-*T. gondii* antibodies, the Indirect Immunofluorescence Test (IFAT) was performed using a cutoff point of 1:64 (Santos et al., 2009) and following the method described by Camargo (1974). The RH strain of *T. gondii* fixed on slides was used as the antigen. Samples showing complete peripheral fluorescence of the tachyzoites were considered positive. Positive samples were subjected to sequential twofold dilutions until negativity was achieved.

### Epidemiological questionnaire

The cattle used in this study were transported to the slaughterhouse upon presentation of the Animal Transit Guide (Guia de Trânsito Animal – GTA), a mandatory document in Brazil that ensures compliance with sanitary regulations and animal traceability. Information on sex, age, and municipality of origin was obtained from the data recorded in the GTAs. Subsequently, the respective owners of the animals were contacted using the contact information present in the GTAs and answered epidemiological questionnaires. The variables analyzed from these questionnaires included: sex (male or female), breed (purebred or crossbreed), age (<1 year, 1-2 wears, >2 years), rearing system (semi-intensive or extensive), purpose of rearing (meat, milk, or mixed), water access (treated or untreated), contact with wildlife (yes or no), contact with other domestic animals (cattle, horses, pigs, sheep, goats, dogs, cats, or poultry), vaccination history (yes-specify which; or no) and history of abortion (yes or no).

### Statistical analysis

Descriptive statistical analysis was used to calculate the frequencies of the results obtained in the IFAT. To identify the factors/consequences associated with the prevalence of anti-*N. caninum* and anti-*T. gondii*, data from epidemiological questionnaires were analyzed in two stages: univariate analysis and multivariate analysis. In the univariate analysis, each independent variable was correlated with the dependent variable (seropositivity), and those with *p-*value ≤ 0.2, according to the Chi-square test (Zar, 1999), were selected for multivariate analysis using multiple logistic regression (Hosmer & Lemeshow, 2000), with a significance level of 5%. To check for any collinearity between the data, a correlation test was applied. If the correlation coefficient was higher than 0.9, one of the variables was eliminated based on the criterion of biological plausibility. The Chi-square and Omnibus tests were used to verify the model’s fit to the parameters. The results were analyzed using GraphPad Prism 8.0.1 software.

## Results

**Figure 1:**
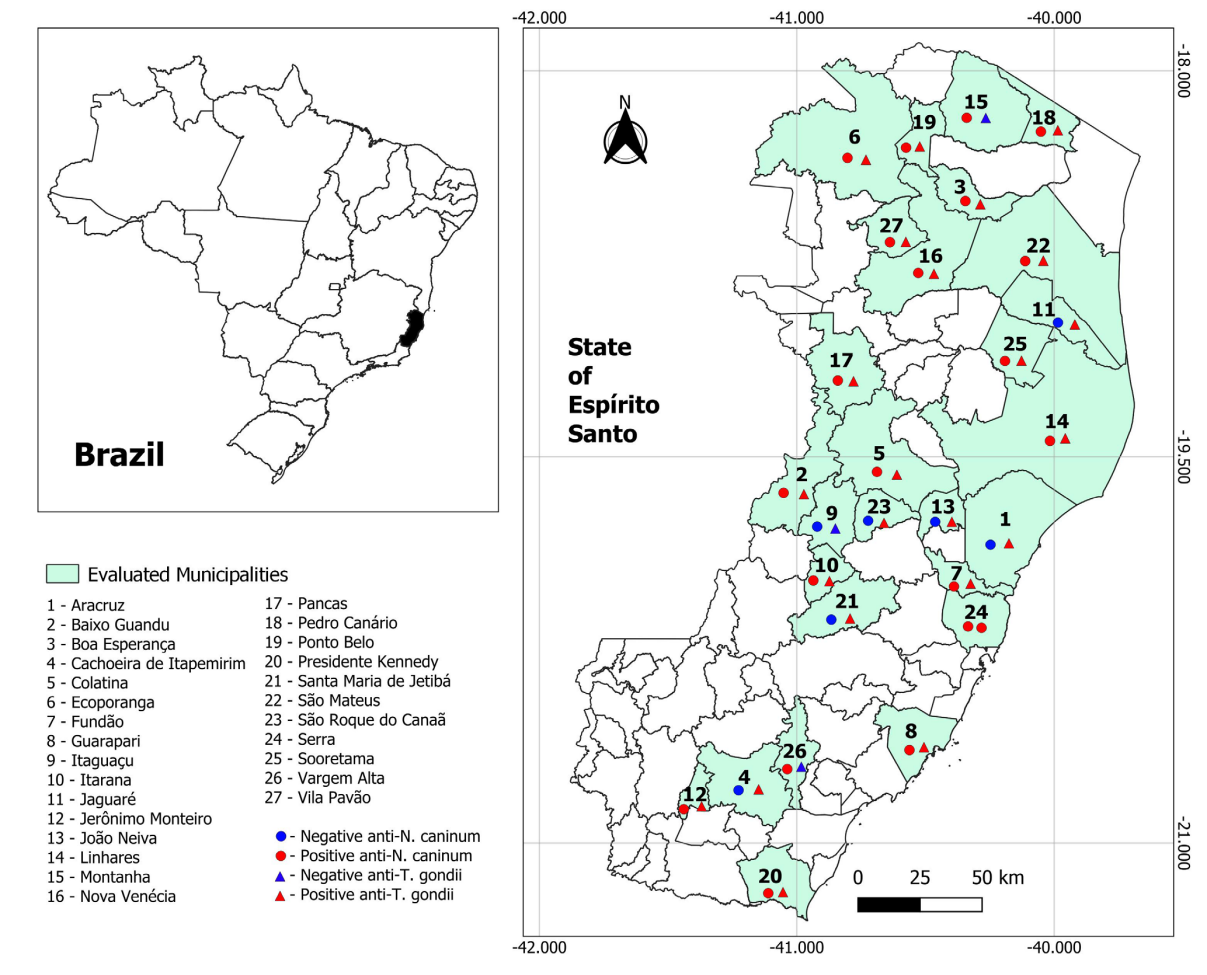
Distribution and positivity of bovine samples tested for anti-*Neospora caninum* and anti-*Toxoplasma gondii* antibodies, by municipality in the state of Espírito Santo, Brazil.

The seroprevalence of anti-*N. caninum* antibodies in cattle from Espírito Santo was 13.2% (79/600), and for anti-*T. gondii* antibodies, it was 12.5% (75/600). The cattle analyzed were sourced from 27 municipalities in the state of Espírito Santo. The geographic locations of the sampled municipalities, indicating whether animals tested positive or negative for anti-*N. caninum* and anti-*T. gondii*, are shown in Figure 1. The number of sampled animals and the percentage of seropositivity by municipality are detailed in Table 1. A high distribution of positivity was observed across the state, with 74.1% (20/27) of municipalities having at least one animal seropositive for *N. caninum* and 88.9% (24/27) for *T. gondii*.

**Table 1:** Distribution of the seroprevalence of cattle infected by *N. caninum* and *T. gondii*, by municipality in the state of Espírito Santo, Brazil.

Municipalities	Samples collected	Positives (%)	
		*N. caninum*	*T. gondii*
Aracruz	6	0 (0)	1 (16.7)
Baixo Guandu	32	6 (18.7)	1 (3.1)
Boa Esperança	11	1 (9.1)	1 (9.1)
Cachoeiro de Itapemirim	7	0 (0)	1 (14.3)
Colatina	10	1 (10)	1 (10)
Ecoporanga	43	6 (13.9)	5 (11.6)
Fundão	12	5 (41.7)	4 (33.3)
Guarapari	14	4 (28.6)	2 (14.3)
Jerônimo Monteiro	7	1 (14.3)	1 (14.3)
Itaguaçu	3	0 (0)	0 (0)
Itarãna	6	1 (16.7)	2 (33.3)
Jaguaré	17	0 (0)	4 (23.5)
João Neiva	8	0 (0)	2 (25)
Linhares	158	22 (13.9)	18 (11.4)
Montanha	6	1 (16.7)	(0)
Nova Venécia	20	4 (20)	4 (20)
Pancas	19	1 (5.3)	4 (21.1)
Pedro Canário	10	1 (1.3)	1 (1.3)
Ponto Belo	33	4 (12.1)	3 (9.1)
Presidente Kennedy	37	5 (13.5)	1 (2.7)
Santa Maria de Jetibá	16	0 (0)	2 (12.5)
São Mateus	44	9 (20.4)	7 (15.9)
São Roque do Canaã	5	0 (0)	1 (20)
Serra	21	3 (14.3)	4 (19.1)
Soretama	38	1 (2.6)	3 (7)
Vargem Alta	10	1 (10)	0 (0)
Vila Pavão	7	2 (28.6)	2 (28.6)

The titers of anti-*N. caninum* ranged from 1:200 to 1:6400, with 1:200 being the most frequent titter, representing 58.2% (46/79) of the samples. For *T. gondii*, antibody titers ranged from 1:64 to 1:1024, with 1:64 being the most common titer, observed in 48% (48/75) of the samples (Table 2).

**Table 2:** Distribution of anti-*N. caninum* and anti-*T. gondii* antibody titers in cattle from the state of Espírito Santo, Brazil.

Positivity for anti-*N. caninum* antibodies						
Titration	1:200	1:400	1:800	1:1600	1:3200	1:6400
Total (%)	46 (58.2)	20 (25.3)	10 (12.7)	-	1 (1.3)	2 (2.5)
Positivity for anti- *T. gondii* antibodies						
Titration	1:64	1:128	1:256	1:512	1:1024	
Total (%)	36 (48)	22 (29.3)	13 (17.3)	3 (4)	1 (1.3)	

In the univariate analysis of factor associated with *N. caninum* infection, the variables that were statistically significant (p < 0.20) included sex, age, abortion history, and contact with dogs. For *T. gondii*, the significant variables (p < 0.20) were age and contact with cats (Table 3). These variables were subjected to multiple logistic regression analysis. The history of abortion was identified as a factor associated with *N. caninum* infection (OR 6.483; CI 6.082-6.884; p < 0.01), while contact with cats was identified as a factor associated with *T. gondii* infection (OR 7.683; CI 7.172-8194; p < 0.005) (Table 4).

**Table 3:** Univariate analysis of factors/consequences associated with *N. caninum* and *T. gondii* infection in cattle from the state of Espírito Santo.

**Variable**	**Total animal**	***Toxoplasma gondii***		***Neospora caninum***	
		**Positives (%)**	**P-value**	**Positives (%)**	**P-value**
Sex					
Male	479	58 (12.1)	0.56	68 (14.2)	< 0.137*
Female	121	17 (14.1)		11 (9.1)	
Age					
< 24 months	138	12 (8.7)	< 0.12*	25 (18.1)	< 0.05*
> 24 months	462	63 (13.6)		54 (11.7)	
Miscarrieges					
No	131	15 (11.4)	0.766	6 (4.6)	< 0.0006*
Yes	469	60 (12.8)		73 (15.6)	
Contact with dogs					
No	172	20 (11.6)	0.7851	16 (9.3)	< 0.0832*
Yes	428	55 (12.8)		63 (14.7)	
Contact with cats					
No	192	9 (4.7)	< 0.0001*	23 (11)	0.6062
Yes	408	66 (16.2)		56 (13.7)	

* Variables that showed a p-value ≤ 0.20 in the chi-square test or Fisher’s exact test.

**Table 4:** Multivariate analysis of factors/consequences associated with *Neospora caninum* and *Toxoplasma gondii* infection in cattle from the state of Espírito Santo.

**Varable**	**Odds ratio**	**Confidence Interval**	**P-value**
*Neospora caninum*			
Miscarriege	6.483	[6.082-6.884]	< 0.01
*Toxoplasma gondii*			
Contact with cats	7.683	[7.172-8.194]	< 0.005

## Discussion

**Figure 2:**
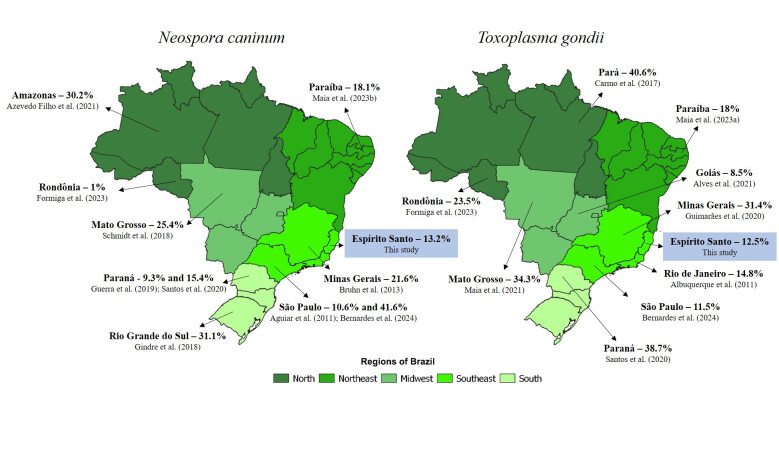
Seroprevalence of anti-*Neospora caninum* and anti-*Toxoplasma gondii* antibodies in cattle in the states of Brazil.

The seroprevalences for *N. caninum* and *T. gondii* were expressive, indicating a widespread distribution of these infections in cattle in the state of Espírito Santo. However, the seroprevalence rates vary across Brazilian states. For anti-*N. caninum* antibodies, in Minas Gerais, a state bordering Espírito Santo, Bruhn et al. (2013) reported a seroprevalence of 21.6%. In São Paulo, also located in the Southeast region of Brazil, Bernardes et al. (2024) found a seroprevalence of 41.6%, while Aguiar et al. (2011) reported 10.6%. This variation was also observed in states from other regions, such as 31.1% in Rio Grande do Sul, 25.4% in Mato Grosso, 18.1% in Paraíba, 9.3% in Paraná, 1% in Rondônia, and 30.2% in Amazonas (Gindri et al., 2018; Schimidt et al., 2018; Guerra et al., 2019; Azevedo et al., 2021; Formiga et al. 2023; Maia et al., 2023b) (Figure 2).

Variations in seroprevalence may occur due to factors such as climate, biome, and the inherent biodiversity of each region. Additionally, the use of different diagnostic methods or cutoff points, as well as differences in the spatial distribution of the parasite, can influence seroprevalence rates (Aguiar et al., 2011; Azevedo et al., 2021).

According to Rossi et al. (2022), the prevalence of *T. gondii* in Brazilian livestock also varies between species and regions. In the present study, the seroprevalence in cattle was lower than that observed in sheep and goats in the same state (Acosta et al., 2021; Vicentini et al., 2024). For anti-*T. gondii* antibodies in cattle, Bernardes et al. (2024) reported 11.5% (75/653) in cattle from the state of São Paulo, Alves et al. (2021) found 8.48% in the state of Goiás, Maia et al. (2023a) reported 18% prevalence in Paraíba, and Albuquerque et al. (2011) reported 14.8% in Rio de Janeiro). However, in Pará, Carmo et al. (2017) described 40.6% seropositivity in cattle. In Mato Grosso, Maia et al. (2021) described 34.27% of seropositivity, while in Rondônia, Formiga et al. (2023) reported 23.5%. In Rio de Janeiro, Frazão-Teixeira & Oliveira (2011) found 49.4% seropositivity. In Minas Gerais, Guimarães et al. (2020) found 31.4% seropositivity (Figure 2). Although cattle are considered resistant to infection, they are not immune, and seroprevalence can be affected by factors such as climate, management and rearing system, which can facilitate the occurrence of infection (Dubey et al., 2004; Maia et al., 2021; Dubey & Julian, 2025). This parasite is zoonotic, and meat from these animals can serve as a source of infection for humans, where the disease may cause clinical signs such as fever, cervical lymphadenopathy, myalgia, fatigue, and ocular, neurological, and reproductive issues (Carmo et al., 2017; Dubey, 2021; Virus et al., 2021; Formiga et al., 2023).

The antibody titers for *N. caninum* showed a wide variation, however, most of the sampled animals presented minimum titers equal to the established cutoff point (1:200), which may indicate a higher chronicity of the infections. This characteristic was also observed in other studies, such as the one conducted by Guerra et al. (2019), which identified a range of 1:100-1:400, with 1:100 being the cutoff point adopted in that study. Sousa et al. (2022) similarly found a predominance of cattle with low antibody titers (up to 1:400). In the present study, it was also possible to identify animals with high antibody titers (up to 1:6400), as noted by Bernardes et al. (2024), who suggested the possibility of a more recent acute infection in these animals.

Similarly, the antibody titers for *T. gondii* also showed a wide variation in this study, with a predominance of animal having titers below 1:256, indicating a possible higher chronicity of the infections. Likewise, Santos et al. (2020) described a variation of 1:64-1:1024 in the anti-*T. gondii* antibody titers in cattle from the State of Paraná, with most animals close to the cutoff point. On the other hand, Alves et al. (2021) found a narrower variation (1:128- 1:256). These data reinforce the hypothesis of lower susceptibility of cattle to *T. gondii* infections. Despite this, Dubey & Julian (2025) described the occurrence of acute, visceral and fatal toxoplasmosis in a calf with the presence of tachyzoites in several tissues and the formation of tissue cysts in the liver. In the present study, it was possible to observe some animals with high titers, reaching up to 1:1024, which may indicate the possibility of a recent acute infection.

The occurrence of abortion was identified as a factor associated with *N. caninum* infection. In a case-control study conducted by Sousa et al. (2022), it was observed that the proportion of animals positive for *N. caninum* was higher among cattle with a history of abortion (OR 4.808; CI 1.919-12.050; p < 0.001) compared to animals with other reproductive disorders (OR 3.178; CI 1.624-6.223; p < 0.001). Similarly, cattle with a history of abortion were more likely to be seropositive than animals that had never aborted (OR 6.483; CI 6.082-6.884; p < 0.01), demonstrating the influence of *N. caninum* on reproductive rates. Santos et al. (2020) identified abortion as a factor associated with infections by both *N. caninum* and *T. gondii*. However, in the present study, only *N. caninum* was associated with this variable, as cattle exhibit a high natural resistance to *T. gondii* (Dubey, 1986). Alternatively, this could be related to the high genetic variability exhibited by *T. gondii* in Brazil, which may influence not only susceptibility but also the occurrence of abortions, as we hypothesize. The virulence of a genotype is directly associated with clinical manifestations, as mild genotypes typically result in subclinical infections, while virulent genotypes can lead to severe clinical signs such as abortion (Dubey & Jones, 2008; Costa et al., 2011; Feitosa et al., 2017a, b; Feitosa et al., 2024; Vilela et al., 2024; Vilela & Feitosa, 2024). There are discrepancies among studies regarding cat contact as a factor associated with *T. gondii* infection (Alves et al., 2021; Rossi et al., 2022; Bernardes et al., 2024). In this research, this variable was significant (OR 7.683; CI 7.172-8.194; p < 0.005). Considering that cattle over 24 months of age showed higher seropositivity (p ≤ 0.2), there is a possibility that this is associated with the horizontal transmission of the parasite through contamination by oocysts shed by infected cats, which contaminate pastures, feeders, and water troughs (Dubey et al., 2004; Maia et al., 2021).

Although it showed statistical significance in the univariate analysis (p ≤ 0.2), contact with dogs was not considered a factor associated with *N. caninum* infection, differing from studies that identified this variable as a risk factor (Ribeiro et al., 2019; Azevedo et al. 2021). Dogs are the definitive hosts of *N. caninum* and contribute to environmental contamination by shedding oocysts in their feces. However, for horizontal transmission to occur, dogs must be infected and actively shedding oocysts in the environment (Lindsay et al., 1999; Palavicini et al., 2007). Moreover, the number of oocysts produced by dogs is lower than that produced by cats, which may explain the results found in the present study (Schares et al., 2005).

Age and sex were not considered factors associated with infection by *N. caninum* or *T. gondii*. Although animals over 24 months old and females showed higher seropositivity (p ≤ 0.2), these findings are consistent with other studies (Venturoso et al., 2021; Bernardes et al., 2024). It is important to consider that seroprevalence increases with the duration of exposure to infection (Azevedo et al., 2021). In this study, the exposure time to potential infection was higher, as females are generally used as breeding stock and take longer to be slaughtered, and most of the animals evaluated were over 24 months old.

## Conclusion

The seroprevalence of *N. caninum* and *T. gondii* cattle in the state of Espírito Santo was expressive and the presence of the protozoans was identified in most of the sampled municipalities. A history of abortions was identified as a factor associated with *N. caninum* infection, while contact with cats was a factor associated with *T. gondii* infection in these animals. It is recommended to include in control and prophylactic strategies the removal of animals with a history of abortion from the breeding line and the restriction of cat access to pastures and production facilities.
